# Optimizing immunotherapy in advanced gastric cancer: established and emerging biomarkers for precision patient selection

**DOI:** 10.3389/fimmu.2026.1775386

**Published:** 2026-07-01

**Authors:** Xianjun Rao, Guodong Huang, Jiaxuan Li, Chunyu Wu, Min Yang, Xianfeng Rao, Zixing Qian, Liji Chen, Yang Yang, Wei Wei

**Affiliations:** 1 Wangjing Hospital, China Academy of Chinese Medical Sciences, Beijing, China; 2 College of Traditional Chinese Medicine, Hubei University of Chinese Medicine, Wuhan, China; 3 The First Affiliated Hospital of Heilongjiang University of Chinese Medicine, Harbin, China; 4 Department of Pediatric Surgery, The Sixth Affiliated Hospital of Harbin Medical University, Harbin, China; 5 Department of Neurosurgery, The First Affiliated Hospital of Harbin Medical University, Harbin, China

**Keywords:** biomarker-guided immunotherapy, circulating tumor DNA, dMMR/MSI-H, gastric cancer, immune checkpoint inhibitors, PD-L1 CPS, perioperative immunotherapy, tumor microenvironment

## Abstract

mmune checkpoint inhibitors have reshaped the therapeutic landscape of advanced gastric and gastroesophageal junction cancer, yet durable benefit remains restricted to biologically selected patient subgroups. Optimizing immunotherapy, therefore, requires a disease-specific, clinically practical, and critically interpreted biomarker framework rather than reliance on any single predictive marker. This review synthesizes established and emerging biomarkers for immune checkpoint inhibitor selection in advanced, metastatic, and perioperative gastric cancer, with emphasis on programmed death-ligand 1 combined positive score, deficient mismatch repair/microsatellite instability-high status, tumor mutational burden, Epstein–Barr virus-associated tumors, HER2-defined combination strategies, tumor microenvironment features, circulating tumor DNA dynamics, and artificial intelligence-assisted models. In addition to summarizing biological rationale, this review critically evaluates trial-based limitations that influence biomarker interpretation, including assay variability, spatial and temporal heterogeneity, dynamic PD-L1 expression, inconsistent CPS thresholds, overlap between TMB-high and MSI-H biology, and the limited prospective validation of exploratory markers. Recent perioperative and neoadjuvant ICI studies are discussed to highlight how early biomarker testing may guide treatment intensification, de-escalation, or trial selection in resectable disease. Emerging evidence supporting TMEscore, longitudinal ctDNA response, and multimodal AI-based prediction is also examined, with an emphasis on the current barriers to routine clinical implementation. Finally, this review proposes a tiered, resource-aware interpretation of biomarkers that distinguishes clinically actionable markers from investigational tools and addresses challenges related to standardization, cost, access, and equity. Overall, biomarker-guided integration of molecular, immune, dynamic, and translational markers is essential to improve patient selection and advance precision immunotherapy in gastric cancer.

## Graphical Abstract

**Figure:**
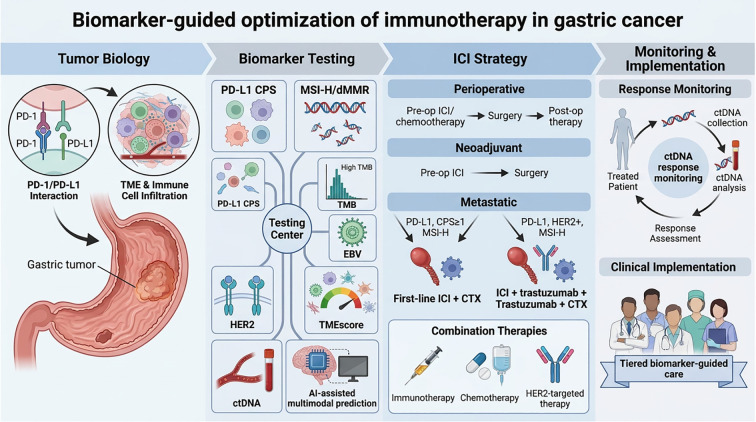
The graphical abstract summarizes how tumor biology, biomarker testing, immune checkpoint inhibitor strategies, and response monitoring are integrated to optimize immunotherapy in gastric cancer. It highlights key biomarkers, including PD-L1 CPS, MSI-H/dMMR, TMB, EBV, HER2, TME features, ctDNA, and AI-assisted prediction, for precision patient selection and clinical implementation

## 1 Introduction

Gastric cancer (GC) is a significant global health burden, the fifth most commonly diagnosed malignant tumor, and the fourth leading cause of cancer-related deaths worldwide. In 2020 alone, over one million new cases of gastric cancer were reported globally, resulting in approximately 769,000 deaths (1). Advanced gastric cancer (AGC), encompassing mid- to late-stage disease characterized by tumor invasion through the full thickness of the gastric wall, is associated with a poor prognosis. Historically, systemic chemotherapy has been the cornerstone of treatment for AGC; however, its clinical benefit remains limited. In recent years, immunotherapy—particularly immune checkpoint inhibitors (ICIs) has emerged as a promising therapeutic strategy for gastric cancer. Clinical studies have demonstrated that patients with specific biological features, including high programmed death ligand-1 (PD-L1) expression, microsatellite instability–high (MSI-H) status, and Epstein–Barr virus (EBV) positivity, derive significant benefit from ICIs, underscoring the critical role of tumor immunogenicity in shaping treatment response (2). Pembrolizumab has been approved by the U.S. Food and Drug Administration (FDA) as a third-line treatment for recurrent or metastatic gastric cancer or adenocarcinoma of the gastroesophageal junction with PD-L1 expression ≥1%. In addition, nivolumab in combination with chemotherapy has been approved as the first-line treatment for advanced or metastatic gastric cancer and gastroesophageal colorectal cancer (GEJC), regardless of PD-L1 status. For patients with human epidermal growth factor receptor 2 (HER2) -positive AGC, the FDA recommends a treatment regimen of combined chemotherapy with the HER2-targeting monoclonal antibody trastuzumab and the programmed death-1 (PD-1) inhibitor pembrolizumab. Despite these advancements, a considerable number of patients still do not respond to immunotherapy, and immune-related adverse events remain a clinical concern. Therefore, it is increasingly important to identify reliable immune-related biomarkers to select patients most likely to benefit from immunotherapy accurately.

GC is a biologically heterogeneous disease, and advancements in genomic mapping have led to the identification of different molecular subtypes. In 2014, the Cancer Genome Atlas (TCGA) classified gastric cancer into four molecular subtypes: EBV-positive tumors, MSI tumors, genome-stable (GS) tumors, and tumors characterized by chromosomal instability (CIN) (3). To complement this framework, the Asian Cancer Research Group (ACRG) has proposed an alternative molecular classification method based on gastric cancer GC) samples from the Asian population. This system also defines four subtypes: the MSI subtype, mainly Lauren’s gut type, is associated with early disease and has the best prognosis; The MSS/EMT subtype, primarily corresponding to Lauren diffuse, is characterized by a younger age of onset, loss of CDH1 expression, and the poorest clinical prognosis. The MSS/TP53+ subtype shows a higher prevalence of EBV infection. MSS/TP53− subtype, HER2 enrichment amplification (4). Although these molecular classifications have enhanced our understanding of the biology and prognosis of gastric cancer, they remain insufficient to predict the response to immunotherapy precisely. Currently, HER2 status and PD-L1 expression are the only biomarkers routinely used to guide the treatment decisions of AGC. HER2-targeted therapy has been shown to improve overall survival in patients with HER2-positive disease significantly (5). However, the predictive value of PD-L1 expression in gastric cancer remains controversial. Other potential biomarkers, including defective mismatch repair (dMMR), tumor mutation burden (TMB), and EBV infection, require further validation in large-scale clinical studies (6, 7). Emerging technologies such as liquid biopsy, single-cell sequencing, and multiplex immunohistochemistry (mIHC) have provided powerful new tools for personalized gas chromatography research and biomarker discovery (8–10).

To maintain a focused and clinically useful scope, this review centers on biomarker-guided immune checkpoint inhibitor strategies for advanced, metastatic, and perioperative gastric/gastroesophageal junction adenocarcinoma, rather than providing a general overview of all gastric cancer treatments. Accordingly, conventional modalities such as surgery, chemotherapy, radiotherapy, and targeted therapy are discussed only when they influence immunotherapy selection, combination strategies, or response assessment. The review also adopts a critical translational perspective by distinguishing biomarkers with established clinical relevance, such as PD-L1 CPS and dMMR/MSI-H status, from emerging or exploratory markers, including TMB, EBV status, TME-based scores, ctDNA dynamics, microbiome features, and AI-assisted models. Particular attention is given to disease-specific limitations in gastric cancer, including assay variability, spatial and temporal heterogeneity, inconsistent cutoffs across trials, overlap among biomarkers, limited prospective validation, cost barriers, and unequal access to molecular testing. This focused structure is intended to support the optimization of immunotherapy through clinically applicable, evidence-based, and resource-aware biomarker interpretation.

## 2 Immunotherapy for gastric cancer

**Figure 1:**
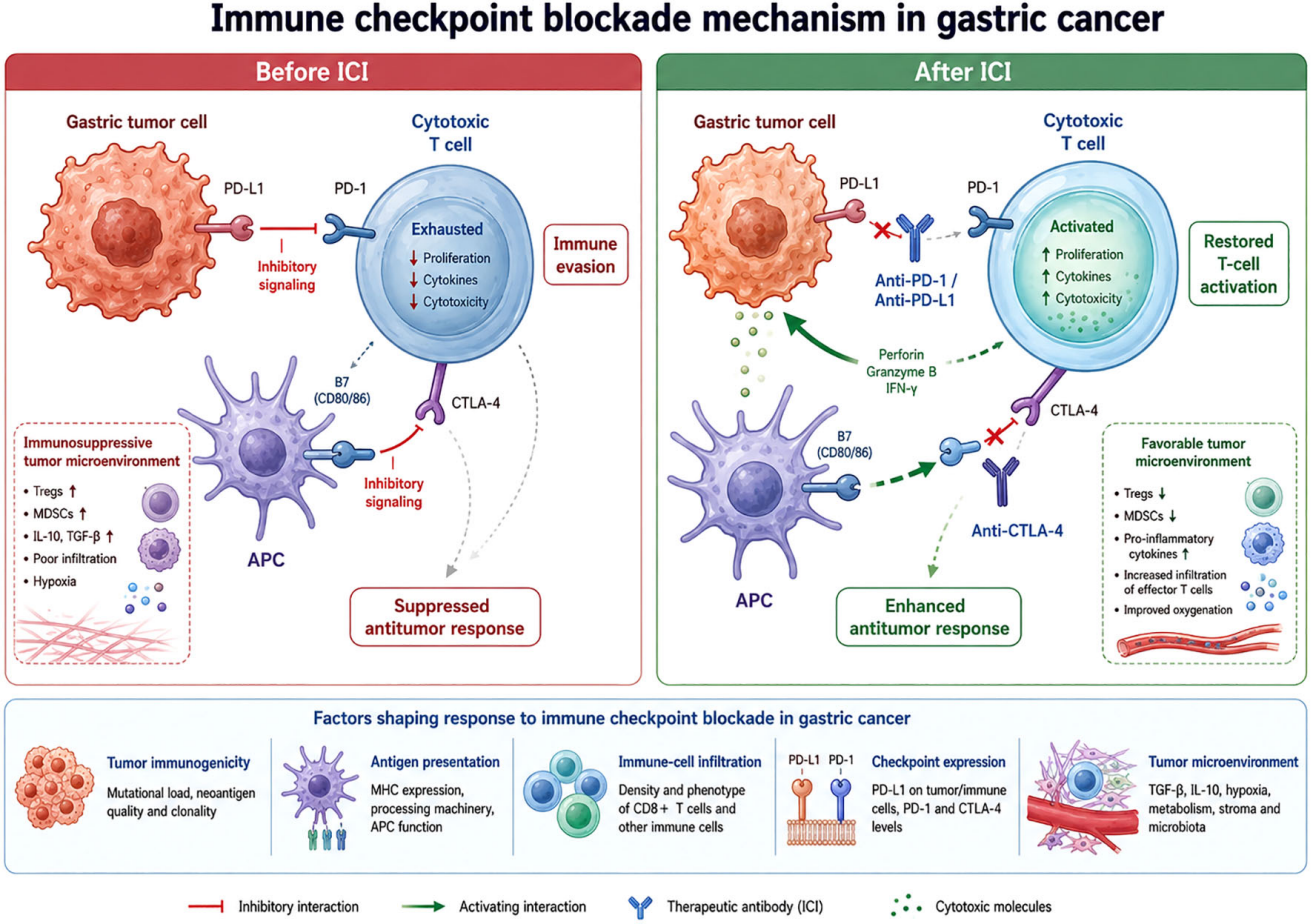
Immune checkpoint blockade mechanism in gastric cancer.

Historically, gastrointestinal malignancies have been treated with surgery, chemotherapy, radiotherapy, or a combination of these methods. Despite these interventions, the overall survival outcomes for many patients remain poor (11). To improve clinical prognosis, treatment strategies are increasingly shifting towards immune-based approaches, that is, activating the host immune system to target cancer cells. This strategy is widely known as immunotherapy (12, 13). Immunotherapy shows considerable promise in the treatment of gastrointestinal cancers, especially in patients in the advanced stage of the disease or those who are resistant to conventional therapies. The main immunotherapy approaches currently used for gastrointestinal malignancies include immune checkpoint inhibitors (ICIs), cytokine therapy, adoptive immunotherapy, cancer vaccines, and chimeric antigen receptor (CAR) T-cell therapy (14). Immune checkpoint inhibitors primarily exert their effects by blocking inhibitory signaling pathways, including programmed cell death protein 1 (PD-1)/programmed cell death ligand 1 (PD-L1) and cytotoxic T-lymphocyte-associated antigen 4 (CTLA-4), thereby enhancing T-cell-mediated anti-tumor immune responses (15). Treatments targeting the PD-1/PD-L1 axis have demonstrated durable anti-tumor activity, particularly in the treatment of drug-resistant malignancies. In the tumor microenvironment, cancer cells exploit PD-1/PD-L1 signaling to evade immune surveillance, leading to impaired T-cell function and increased regulatory T-cell activity. Blocking this pathway restores immune-mediated antitumor activity (16, 17). The mechanistic basis of immune checkpoint blockade in gastric cancer is summarized in **Figure 1**.

The figure illustrates how PD-1/PD-L1 and CTLA-4 signaling suppress T-cell activation and promote immune evasion in gastric cancer. Blocking these checkpoints with immune checkpoint inhibitors restores cytotoxic T-cell activity, enhances antitumor immune responses, and reshapes the tumor microenvironment.

PD-1inhibitors, including pembrolizumab, nivolumab, and avelumab, have been approved for clinical application in gastrointestinal malignancies. Several studies have confirmed their therapeutic effect (18). In addition, therapies targeting CTLA-4 (which inhibits T cell activation by binding to CD80/CD86 on antigen-presenting cells) have been approved for the treatment of gastrointestinal cancers. Since PD-1/PD-L1 and CTLA-4 regulate different stages of the immune response, combined inhibition of these pathways has been proven to improve clinical outcomes (19, 20). The therapeutic response is influenced by multiple factors, including the burden of tumor mutations, the presence of tumor-infiltrating lymphocytes, and the expression of immune checkpoint receptors (21). However, the significant heterogeneity among patients complicates the standardization of treatment. Therefore, identifying patients most likely to benefit from immune checkpoint suppression remains a considerable challenge, underscoring the urgent need for more accurate predictive biomarkers to inform decisions about personalized immunotherapy (22, 23).

## 3 Gastric cancer: classification, risk factors, and diagnostic strategies

### 3.1 Classification of gastric cancer

Gastric cancer (GC) is classified based on histopathological features, anatomical origin, and disease progression. Among these systems, the Lauren classification remains one of the most commonly used due to its simplicity and long-term clinical relevance. **Table 1** illustrates that this classification categorizes gastric cancer GC) into four subtypes: well-differentiated, poorly differentiated, mixed, and uncertain. Each subtype has its own clinical and pathological features. The World Health Organization (WHO) released a new classification framework in 2010. This is now considered the most complete classification system for gastric tumors (24). The system also talks about some less well-known types of stomach tumors, in addition to gastric adenocarcinoma. There are different types of gastric adenocarcinoma (GAC), such as tubular carcinoma, papillary carcinoma, micropapillary carcinoma, mucinous type, mucoepidermoid carcinoma, parietal cell carcinoma, mixed type, low-viscosity carcinoma (including signed-ring cell carcinoma), medullary carcinoma, hepatic adenocarcinoma, and Paneth cell carcinoma (25, 26).

**Table 1:** Trial-based comparison of PD-L1 testing approaches and biomarker limitations in gastric/gastroesophageal junction cancer.

Trial	Treatment setting	ICI strategy	PD-L1 assay/scoring approach	Key biomarker implication or limitation
KEYNOTE-061	Previously treated advanced gastric/GEJ cancer	Pembrolizumab vs paclitaxel	PD-L1 CPS ≥1 using 22C3	CPS ≥1 was insufficient as a strong predictor because pembrolizumab did not significantly improve OS in the primary CPS ≥1 population, suggesting that low PD-L1 thresholds may dilute biomarker-enriched benefit (60).
KEYNOTE-062	First-line advanced gastric/GEJ cancer	Pembrolizumab ± chemotherapy vs chemotherapy	PD-L1 CPS ≥1 and CPS ≥10 using 22C3	Pembrolizumab showed greater clinical relevance in higher CPS groups, indicating that the predictive value of PD-L1 is threshold-dependent rather than binary (61).
CheckMate-649	First-line advanced gastric/GEJ/esophageal adenocarcinoma	Nivolumab plus chemotherapy vs chemotherapy	PD-L1 CPS ≥5 as the primary enrichment population	Survival benefit was clearest in CPS ≥5 tumors, supporting CPS ≥5 as a clinically useful enrichment cutoff, but limiting direct comparison with pembrolizumab trials using CPS ≥1 or CPS ≥10 (51).
KEYNOTE-859	First-line HER2-negative advanced gastric/GEJ cancer	Pembrolizumab plus chemotherapy vs chemotherapy	PD-L1 CPS ≥1 and CPS ≥10 using 22C3	Benefit was observed in the overall population and PD-L1 subgroups, but the magnitude of benefit varied by CPS level, reinforcing the need for cutoff-specific interpretation (52).
KEYNOTE-811	First-line HER2-positive advanced gastric/GEJ cancer	Pembrolizumab plus trastuzumab and chemotherapy	HER2 positivity with PD-L1 CPS-based interpretation	This trial illustrates biomarker interaction, in which HER2 defines the targeted backbone, and PD-L1 helps refine immunotherapy benefit, making PD-L1 interpretation dependent on molecular context (62).
RATIONALE-305	First-line advanced gastric/GEJ adenocarcinoma	Tislelizumab plus chemotherapy vs chemotherapy	TAP ≥5% primary PD-L1-positive population	Use of TAP rather than CPS demonstrates that different PD-L1 scoring algorithms can define distinct biomarker-positive populations, complicating comparisons with CPS-based trials (63).
ORIENT-16	First-line unresectable gastric/GEJ cancer	Sintilimab plus chemotherapy vs chemotherapy	PD-L1 CPS ≥5 and the overall population	Benefits in both CPS ≥5 and all-randomized populations support PD-L1 enrichment but also show that PD-L1 alone does not fully explain ICI benefit (54).
PD-L1 assay and heterogeneity studies	Biomarker validation context	Not treatment-specific	22C3, 28-8, SP263, CPS/TAP comparison	Analytical differences among assays and spatial/temporal heterogeneity of PD-L1 expression and TMB may affect patient classification, especially when small biopsies or archival tissue are used (64).

Gastric cancer is generally categorized based on tumor location into cardia cancer, which occurs in the stomach or at the esophagus-stomach junction, and non-cardia cancer, which manifests in the distal portion of the stomach. There are significant differences in the epidemiology and risk factors between these two types. The rise in the number of cardia and gastroesophageal junction (GEJ) tumors is linked to the increase in the number of people who are obese and have gastroesophageal reflux disease (27). Gastric cancer frequently exhibits rapid progression, resulting in a significant percentage of patients being diagnosed at an advanced stage. There are four stages of GC in the clinic: 0 to IV, with intermediate stages IA, IB, IIA, IIB, IIIA, IIIB, and IIIC. Stage 0, also known as carcinoma *in situ*, is limited to the epithelium. Stage II disease entails a more profound infiltration of the gastric wall, along with lymph node involvement. In the third stage, the tumor spreads from the muscle layer to the surrounding connective tissue and nearby organs. The fourth stage is when the cancer spreads to other parts of the body, like the stomach and other organs. After stage II, the chances of survival dropped sharply. It was reported that the 5-year survival rate fell from about 61–63% in stage IIIA to 30–35% in stage IIIC (28).

### 3.2 Risk factors and causes

The interplay of various elements, including infectious agents, genetic predisposition, and environmental and lifestyle exposures, facilitates the onset of gastric cancer. the leading causes are Helicobacter pylori (H. pylori) infection, Epstein-Barr virus (EBV), genetic syndrome, and environmental factors. About half of the people in the world have H. pylori, and almost 2% of those who do get stomach cancer. The World Health Organization classifies these bacteria as a class 1 carcinogen. Finding and eliminating H. pylori early on can reverse precancerous lesions in the stomach and significantly reduce the risk of stomach cancer. Cytotoxin-associated gene A (CagA) is one of the most important virulence factors of Helicobacter pylori. It helps peptic ulcers form and then turn into cancer. Caga-positive strains were identified in nearly all Asian patients and about 70% of American patients. Helicobacter pylori causes chronic inflammation by releasing peptidoglycan into host cells. This causes the body to produce more inflammatory mediators, such as interleukin-8 (IL-8) and cyclooxygenase (COX). Moreover, the VacA toxin released by Helicobacter pylori inhibits T-cell function and promotes the development of precancerous lesions. Early treatment before ulcers form can significantly lower the risk of stomach cancer (29).

About 10% of stomach cancer cases are caused by the Epstein-Barr virus. EBV selectively infects cells that exhibit elevated levels of CD21 receptors (30). IgA-coated EBV virus particles can enter epithelial cells through endocytosis, which IgA receptors help with. The gH/gL ligand facilitates viral entry into the cell and integration, and the gH/gL/gB complex binds to the epithelial membrane. EBV also interacts with the host β2 integrin and BMFR2, thereby exacerbating infection. These interactions between molecules are what cause EBV-related gastric cancer to happen. Genetic factors contribute to roughly 1-3% of gastric cancer cases and can be categorized into three principal genetic syndromes. Hereditary diffuse gastric cancer (HDGC) is an autosomal dominant genetic disorder primarily resulting from mutations in genes that encode the junction components of epithelial adhesives, specifically CDH1 and CTNNA1, which encode E-cadherin and α-catenin, respectively (31). Gastric adenocarcinoma and proximal gastric polyposis (GAPPS) is another autosomal-dominant syndrome linked to germline mutations in the 1B region of the APC gene promoter. GAPPS is marked by polyposis of the basal gland that is only found in the gastric body and fundus. Most of the time, patients do not have Helicobacter pylori infection (32). Familial gastrointestinal carcinoma (FIGC) and familial diffuse gastric cancer (FDGC) are additional genetic syndromes, although the precise genetic mechanisms underlying these conditions remain inadequately understood (33).

Environmental and lifestyle factors also play a significant role in the development of gastric cancer. Eating a lot of salt, hot foods, and late-night meals, having trouble sleeping and waking up, smoking, and drinking too much alcohol all raise the risk of gastric cancer. Eating red meat regularly was also linked to a higher risk of getting sick. Long-term use of PPIs may also change the diversity of microbes in the stomach, which could lead to cancer-causing processes (34). To develop effective prevention and early detection plans, it is essential to identify modifiable risk factors.

### 3.3 Diagnostic and screening methods

For effective treatment of gastric cancer, it is essential to get an early and accurate diagnosis. This is especially true for detecting advanced or diffuse disease and distinguishing intestinal from diffuse histological subtypes. Currently, a range of diagnostic techniques has been implemented, including upper gastrointestinal barium meal radiography, esophagogastroduodenoscopy, endoscopic ultrasound, computed tomography, and fluorodeoxyglucose positron emission tomography (FDG PET). Among these techniques, magnifying narrow-band imaging (M-NBI) has emerged as a valuable diagnostic tool, enabling the accurate visualization and characterization of gastric lesions. Recent advancements in M-NBI have enhanced diagnostic accuracy and facilitated the judicious implementation of therapeutic interventions, including endoscopic mucosal dissection (35). Numerous serum biomarkers have been suggested to detect individuals at heightened risk for gastric cancer. As atrophic gastritis worsens, serum pepsinogen I (PGI) levels decrease, whereas pepsinogen II (PGII) levels remain relatively stable. As a result, a lower PGI/II ratio is linked to a higher risk of developing gastric cancer, and a higher ratio is linked to a lower risk. Adding a serum pepsinogen test to a Helicobacter pylori serology test can further enhance predictive accuracy. People with a low PGI or PGI/II ratio and negative Helicobacter pylori antibodies may be the most at risk and may have severe gastric atrophy.

Chronic inflammation and atrophic gastritis resulting from Helicobacter pylori infection may result in diminished gastric acid secretion, indicating that low serum gastric acid levels could be linked to an elevated risk of gastric cancer. Gastric secretory element and parietal cell antibody (APCA) are two additional biomarkers that may help identify high-risk groups (36). As a timely and accurate diagnosis is essential for effective patient management, the focus now shifts to assessing treatment strategies, encompassing both conventional and novel targeted methodologies.

## 4 Biomarker-guided integration of immunotherapy with current treatment strategies in gastric cancer

**Figure 2:**
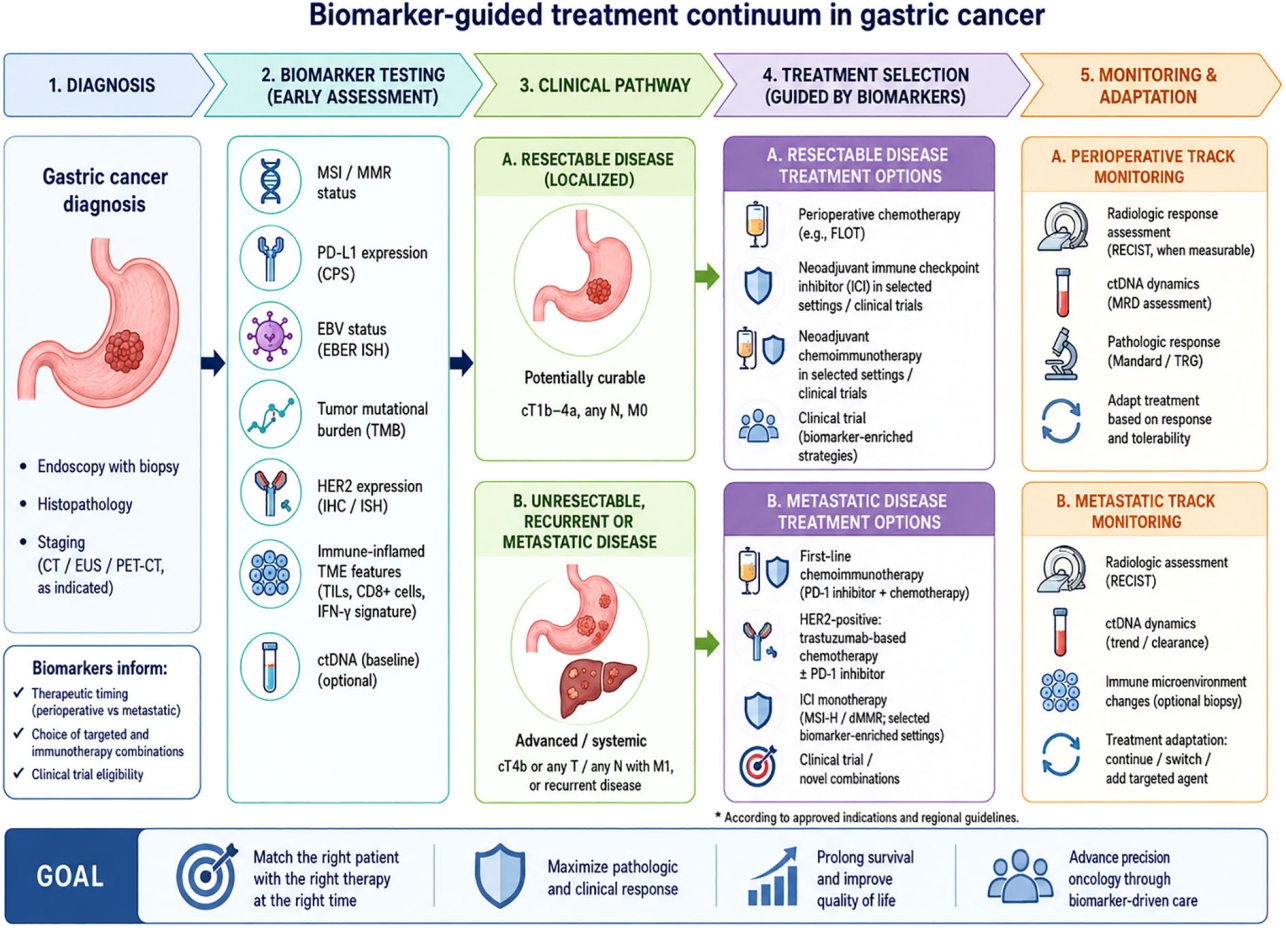
Biomarker-guided treatment continuum in gastric cancer.

The management of gastric and gastroesophageal junction adenocarcinoma has evolved from a primarily stage-based treatment model to an increasingly biomarker-guided strategy in which surgery, chemotherapy, targeted therapy, radiotherapy, and immune checkpoint inhibitors are selected and sequenced based on tumor biology, treatment intent, and expected immune responsiveness. Therefore, treatment modalities should not be discussed as isolated options in the context of immunotherapy-focused precision medicine; rather, they should be interpreted as clinical backbones that can enhance, complement, or refine ICI-based strategies. In resectable disease, curative surgery remains essential, but perioperative systemic therapy has become a major determinant of long-term outcomes. The FLOT4 trial established perioperative fluorouracil, leucovorin, oxaliplatin, and docetaxel as a standard chemotherapy backbone for fit patients with locally advanced resectable gastric or gastroesophageal junction adenocarcinoma (37). However, biomarker-defined subgroups are increasingly challenging the assumption that all patients benefit equally from chemotherapy-based perioperative treatment. In particular, dMMR/MSI-H tumors show high neoantigen load, immune-cell infiltration, and strong sensitivity to immune checkpoint blockade. At the same time, available evidence suggests that the benefit of perioperative chemotherapy in this subgroup may be limited or inconsistent (38). This biological distinction supports early molecular testing at diagnosis, even in potentially resectable disease, because MSI/MMR status, PD-L1 expression, EBV status, HER2 expression, immune-inflamed tumor microenvironment features, and emerging liquid biopsy markers may influence whether patients are best treated with chemotherapy alone, chemoimmunotherapy, dual immune checkpoint blockade, or biomarker-enriched clinical trial approaches. This biomarker-guided treatment continuum, from diagnosis through treatment selection to response monitoring, is illustrated in **Figure 2**.

The figure presents a treatment continuum from diagnosis and early biomarker testing to clinical pathway selection, biomarker-guided treatment, and response monitoring. It shows how biomarkers such as MSI/MMR, PD-L1 CPS, EBV, TMB, HER2, TME features, and ctDNA guide treatment decisions in resectable, unresectable, recurrent, and metastatic gastric cancer.

### 4.1 Perioperative and neoadjuvant immunotherapy strategies in biomarker-defined resectable disease

In localized and locally advanced gastric cancer, perioperative and neoadjuvant immunotherapy are attractive because the intact primary tumor, preserved tumor-draining lymph nodes, and ongoing antigen presentation may support stronger T-cell priming than postoperative therapy alone. This rationale is particularly relevant for dMMR/MSI-H tumors, where abundant mutation-associated neoantigens enhance immune recognition. In the GERCOR NEONIPIGA phase II study, neoadjuvant nivolumab plus ipilimumab followed by surgery and adjuvant nivolumab produced a high pathological complete response rate in localized dMMR/MSI-H gastric or gastroesophageal junction adenocarcinoma, supporting MSI/MMR status as one of the most clinically meaningful biomarkers for selecting patients for neoadjuvant immune checkpoint blockade (39). Similarly, the INFINITY study showed promising activity of chemotherapy-free tremelimumab plus durvalumab as a neoadjuvant or non-operative management strategy in MSI-H resectable gastric or gastroesophageal junction adenocarcinoma, suggesting that selected patients with highly immunogenic tumors may eventually be considered for organ-preserving or surgery-de-escalation approaches, although this remains investigational and requires longer follow-up and larger validation cohorts (40). Beyond MSI-H disease, perioperative chemoimmunotherapy is being evaluated in broader resectable populations. The DANTE/IKF-s633 trial demonstrated that adding atezolizumab to perioperative FLOT was feasible and improved pathological regression parameters, with stronger responses observed in tumors with higher PD-L1 expression or MSI-H status, indicating that immune-enriched molecular profiles may identify patients more likely to benefit from perioperative ICI intensification (41). The PANDA study also supported the biological rationale for neoadjuvant immunotherapy plus chemotherapy by showing that baseline immune features, including activated T-cell infiltration, may be associated with pathological response in resectable gastric and gastroesophageal junction cancer (42). However, perioperative immunotherapy should not yet be considered uniformly effective across all patients. KEYNOTE-585 showed that adding pembrolizumab to perioperative chemotherapy increased pathological complete response but did not clearly establish a definitive event-free survival advantage at interim analysis, emphasizing the need for biomarker-refined selection rather than indiscriminate use of perioperative ICIs (43). In contrast, the phase III MATTERHORN trial reported improved event-free survival with perioperative durvalumab plus FLOT compared with FLOT alone in resectable gastric or gastroesophageal junction adenocarcinoma, suggesting that perioperative chemoimmunotherapy is becoming a clinically important strategy while still requiring interpretation according to PD-L1 status, MSI/MMR status, tumor stage, pathological response, and recurrence risk (44). The negative ATTRACTION-5 trial, in which adjuvant nivolumab plus chemotherapy did not support routine addition of postoperative nivolumab after D2 gastrectomy for stage III gastric or gastroesophageal junction cancer, further reinforces that the timing of immunotherapy matters and that neoadjuvant or perioperative immune activation may be biologically more effective than postoperative escalation alone in unselected patients (45). Together, these findings indicate that biomarker testing should be performed before treatment initiation, not only after recurrence, because perioperative decision-making increasingly depends on identifying immune-responsive tumors before surgery.

The expanding perioperative and neoadjuvant ICI literature also indicates that biomarker interpretation in resectable gastric cancer should move beyond metastatic-disease assumptions. MSI-H/dMMR currently represents the most biologically and clinically actionable marker because it is associated with a favorable prognosis, reduced reliance on conventional chemotherapy in some resectable cohorts, and strong sensitivity to PD-1 blockade (46, 47). PD-L1 expression may be enriched for ICI benefit, but its predictive value remains limited by spatial heterogeneity, assay variability, and scoring differences; therefore, it should be interpreted alongside MSI/MMR status and immune contexture rather than as a stand-alone marker (48). TMB-H may support immune sensitivity by reflecting increased neoantigen load. Still, in gastric cancer, its value partly overlaps with MSI-H biology and requires cautious interpretation in microsatellite-stable tumors (49). EBV-positive tumors are also relevant because they frequently show PD-L1 enrichment and immune-inflamed features, although prospective perioperative validation remains insufficient (50). Accordingly, future perioperative and neoadjuvant ICI strategies should integrate MSI/MMR status, PD-L1 expression, TMB, EBV status, and TME-based immune profiling to identify patients most likely to achieve durable pathological and survival benefit.

### 4.2 Biomarker-guided combination therapy in unresectable, recurrent, and metastatic disease

In unresectable, recurrent, or metastatic gastric cancer, immunotherapy is most established as part of first-line combination treatment, and biomarker interpretation is central to estimating the magnitude of benefit. For HER2-negative advanced disease, chemotherapy remains the cytotoxic backbone, but the addition of PD-1 blockade has changed the standard therapeutic framework. CheckMate-649 demonstrated that nivolumab plus chemotherapy improved survival compared with chemotherapy alone, with the clearest benefit in patients whose tumors had a PD-L1 combined positive score ≥5, supporting PD-L1 CPS as a clinically useful but imperfect enrichment biomarker (51). KEYNOTE-859 similarly showed that pembrolizumab plus chemotherapy improved outcomes in HER2-negative advanced gastric or gastroesophageal junction adenocarcinoma, reinforcing chemoimmunotherapy as a major first-line option while also highlighting the need to interpret benefit according to PD-L1 expression, MSI/MMR status, tumor burden, performance status, and expected toxicity (52). Additional phase III studies, including RATIONALE-305 and ORIENT-16, further support the survival benefit of combining PD-1 blockade with chemotherapy in advanced gastric or gastroesophageal junction cancer, particularly in PD-L1-positive populations, but differences in antibodies, scoring systems, cutoffs, geographic populations, and assay platforms illustrate why standardization of biomarker testing remains essential for clinical translation (53, 54). In HER2-positive disease, KEYNOTE-811 provides a clear example of biomarker-driven combination therapy: HER2 positivity defines the trastuzumab-based targeted backbone, while pembrolizumab is added to trastuzumab and chemotherapy, particularly in PD-L1–positive tumors, demonstrating how oncogenic and immune biomarkers can be integrated into the same treatment algorithm (55). In this setting, radiotherapy and palliative interventions should be discussed primarily in relation to immunotherapy rather than as independent treatment categories. Radiotherapy may increase tumor antigen release, promote inflammatory signaling, and potentially enhance immune recognition. At the same time, palliative procedures remain important for symptom relief and for maintaining performance status, enabling patients to receive systemic therapy. Finally, circulating tumor DNA is emerging as a dynamic biomarker that can complement tissue-based testing by monitoring early molecular response, residual disease, clonal evolution, and resistance during ICI-based therapy. Recent studies have shown that early ctDNA decline during chemotherapy plus immune checkpoint inhibition is associated with improved response and survival, suggesting that liquid biopsy may help guide treatment continuation, escalation, or switching before radiological progression becomes evident (56, 57). Thus, the clinical role of treatment modalities in advanced gastric cancer should be framed as a biomarker-guided continuum in which PD-L1 CPS, dMMR/MSI-H status, TMB, EBV status, HER2 expression, immune-cell infiltration, and ctDNA dynamics collectively inform patient selection, treatment combination, therapeutic timing, and response monitoring.

## 5 Biomarkers predicting response to immune checkpoint inhibitors

Although the response to immunotherapy varies widely among patients, predictive biomarkers provide valuable tools for identifying patients most likely to benefit from immune checkpoint inhibitor (ICI) therapy and for optimizing combination therapy regimens (49). The following section discusses some well-known and recently discovered biomarkers for predicting response to immunotherapy.

### 5.1 PD-L1 expression: clinical value and limitations as a predictive biomarker

Programmed cell death protein 1 (PD-1, CD279) is mainly expressed on activated T cells and B cells and is crucial for maintaining immune tolerance at the central and peripheral levels. PD-1 inhibits peripheral T cell activation through its interactions with its ligands, programmed cell death ligand 1 (PD-L1, B7-H1) and programmed cell death ligand 2 (PD-L2, B7-DC) (58). In the tumor microenvironment, PD-L1 is mainly expressed on tumor cells and immune cells. Genetic characteristics related to T cell activation and the local release of interferon-γ (IFN-γ) by T cells can both increase PD-L1 expression (59). The U.S. Food and Drug Administration (FDA) has approved PD-L1 expression as an adjunctive diagnostic biomarker to aid in the selection of immunotherapy, given its immunomodulatory effect and its association with variability in clinical responses.

#### 5.1.1 Trial-based limitations of PD-L1 as a predictive biomarker

Although the PD-L1 combined positive score (CPS) is the most widely used biomarker for selecting patients with advanced gastric or gastroesophageal junction adenocarcinoma for immune checkpoint inhibitor therapy, its clinical interpretation remains limited by assay variability, intratumoral and intertumoral heterogeneity, dynamic changes during treatment, and inconsistent cutoff values across pivotal trials. Several studies used different antibodies, scoring systems, and enrichment thresholds, including CPS ≥1, CPS ≥5, CPS ≥10, and tumor area positivity (TAP) ≥5%, making direct cross-trial comparison difficult. Moreover, PD-L1 expression may differ between primary and metastatic lesions. It may change after chemotherapy or immune pressure, meaning that a single pretreatment biopsy may not fully represent the evolving immune status of the tumor. Therefore, PD-L1 should be interpreted as an enrichment marker rather than a definitive stand-alone predictor, and its predictive value is strongest when integrated with MSI/MMR status, TMB, EBV status, HER2 status, and broader tumor microenvironment features in **Table 1**.

### 5.2 Tumor microenvironment–associated biomarkers

The tumor microenvironment (TME) has a significant impact on clinical outcomes, treatment responses, and tumor development (65). In addition to malignant cells, the TME contains many non-malignant cell types, including fibroblasts, endothelial cells, neurons, adipocytes, and innate and adaptive immune cells. It also includes soluble components, such as growth factors, cytokines, chemokines, and extracellular vesicles, as well as non-cellular components, including the extracellular matrix (66). The composition of ME significantly affects therapeutic outcomes and prognosis in patients. For instance, neutrophils can inhibit lymphocyte-mediated immune responses by secreting cytokines and chemokines, which may explain the correlation between high neutrophil counts and low-level immunotherapy outcomes (67). Tumor-infiltrating lymphocytes (TIL) play a crucial role in anti-cancer immune activity, particularly CD3+ and CD8+ T cells (68). Many studies will use the neutrophil-to-lymphocyte ratio (NLR) as a predictor of the immune response to treatment. A greater NLR is typically associated with an increased number of tumor-associated neutrophils or a malignant tumor signal based on immune cells; neutrophils can either promote or prevent tumor growth (69). For patients receiving immune checkpoint blockade (ICB) treatment, the NLR may be a useful prognostic biomarker, as clinical studies have shown that elevated NLR at baseline and during treatment is closely associated with adverse clinical outcomes (70).

Recent gastric cancer-specific evidence indicates that composite TME-based models may improve the prediction of ICI benefit beyond single biomarkers such as MSI status or PD-L1 CPS. Zeng et al. developed a TMEscore model for advanced gastric cancer by integrating immune and stromal features of the tumor microenvironment. They reported that this score improved patient stratification for checkpoint immunotherapy compared with conventional biomarkers alone (71). More recently, the prospective TIMES001 trial validated an RNA-based TMEscore assay in advanced gastric cancer and demonstrated stronger predictive performance than MSI status and CPS, supporting its potential clinical value in identifying patients most likely to derive a survival benefit from immunotherapy (72). This is biologically reasonable because TMEscore captures the broader immune contexture of the tumor, including immune activation, stromal composition, and suppressive microenvironmental features. In contrast, MSI and CPS reflect only selected aspects of tumor immunogenicity. However, TMEscore is not yet ready for routine clinical use because assay platforms, gene panels, tissue-processing methods, scoring algorithms, and cutoff values remain insufficiently standardized. In addition, most available data still require broader prospective validation across different treatment lines, ethnic populations, metastatic sites, and ICI-combination regimens. Therefore, TMEscore should currently be regarded as a promising translational biomarker rather than a fully established clinical test, with its greatest near-term value lying in integration with MSI/MMR status, PD-L1 CPS, TMB, EBV status, and spatial immune profiling to optimize biomarker-guided immunotherapy in metastatic gastric cancer (73).

### 5.3 Mismatch repair deficiency and microsatellite instability

Mismatch repair defects (dMMR) and microsatellite instability are recognized across a wide variety of tumor types as immunotherapy biomarkers (74). The MMR system, which includes MLH1, MSH2, MSH6, and PMS2, maintains genomic integrity by correcting errors during DNA replication. The loss of MMR function leads to high-frequency microsatellite instability (MSI-H). At the same time, complete MMR is associated with microsatellite stability (MSS) or low-frequency MSI (75). With the FDA’s approval of pembrolizumab for the tissue-uncertain dMMR/MSI-H solid tumors, more and more people are paying attention to the application of this biomarker in gastric cancer (76, 77). Clinical trials, such as Keynote-059 and Keynote-158, have demonstrated that response rates and survival outcomes in patients with dMMR/MSI-H gastric cancer treated with pembrolizumab have been significantly improved (78, 79). The subgroup analysis of Keynote-062 further confirmed that MSI-H patients receiving immunotherapy had higher OS than those receiving chemotherapy.

It is worth noting that significant Phase III trials, including Orient 16 and CheckMate-649, have reported survival benefits in the overall population, with more pronounced effects in the dMMR/MSI-H subgroup, suggesting that even MSS patients may benefit under certain conditions. However, the variability of MMR/MSI detection methods (including immunohistochemistry, PCR-based detection, and next-generation sequencing) has led to inconsistencies in classification (80). Integrating multiple detection methods can enhance diagnostic accuracy and improve patients’ choices.

### 5.4 Tumor mutational burden: gastric cancer–specific interpretation and limitations

Tumor mutational burden (TMB) is biologically attractive as an immunotherapy biomarker because a higher number of somatic mutations may increase neoantigen formation and enhance immune recognition; however, its clinical use in gastric cancer requires more disease-specific caution than a pan-tumor interpretation allows. In advanced gastric and gastroesophageal junction adenocarcinoma, exploratory analyses from KEYNOTE-061 and KEYNOTE-062 suggested that higher tissue TMB may be associated with improved outcomes after pembrolizumab-based therapy. Still, these findings were insufficient to establish TMB as an independent routine biomarker, as its predictive effect partly overlaps with dMMR/MSI-H biology (81, 82). This limitation is particularly important because MSI-H gastric tumors are frequently hypermutated, and the apparent benefit of TMB-H may be driven by the same immune-sensitive subgroup rather than by TMB alone. In KEYNOTE-062, the clinical utility of TMB was attenuated after excluding MSI-H tumors, suggesting that TMB-H should be interpreted with caution in microsatellite-stable disease. Similarly, exploratory biomarker analyses from CheckMate-649 showed that hypermutated/TMB-high biology strongly overlapped with MSI-H status, with all TMB-high tumors in that analysis also classified as MSI-H, further limiting conclusions about the independent predictive value of TMB in gastric cancer (83). Another unresolved issue is the absence of a validated gastric cancer–specific TMB cutoff. Although the pan-tumor pembrolizumab approval used a threshold of ≥10 mutations/Mb, this cutoff cannot be applied to gastric cancer without adjustment, as TMB values vary across sequencing platforms, panel sizes, bioinformatics pipelines, tumor purity, and disease context. A Korean retrospective study proposed 13.31 mutations/Mb as an optimal cutoff for predicting response to PD-1 inhibitor monotherapy in advanced gastric cancer. Still, the cohort was small, single-center, and based on salvage-line treatment, limiting its generalizability to first-line chemoimmunotherapy, perioperative immunotherapy, or non-Asian populations (84). More recent gastric cancer data suggest that qualitative features of mutation burden, such as insertion/deletion rate and frameshift-related neoantigen potential, may complement TMB and better identify immune-responsive microsatellite-stable tumors. Still, these findings also require prospective validation (85). Therefore, in gastric cancer, TMB should be considered a complementary and exploratory biomarker rather than a stand-alone determinant of ICI eligibility. For treatment optimization, TMB is most informative when interpreted alongside MSI/MMR status, PD-L1 CPS, EBV status, HER2 status, and tumor microenvironment features. At the same time, future prospective trials should establish reproducible GC-specific thresholds across treatment settings and testing platforms.

### 5.5 Epstein–Barr virus–associated gastric cancer

Epstein-Barr virus-related gastric cancer represents a unique molecular subtype characterized by strong immune activation (2). EBV-positive tumors exhibited increased PD-L1 expression, enhanced interferon-γ (IFN-γ) signaling, and intense infiltration by CD8+ tumor-infiltrating lymphocytes (TILs), indicating greater sensitivity to ICIs (50). Clinical observations support this hypothesis. Fuchs et al. reported the objective responses of all EBV-positive gastric cancer patients treated with psimumab. At the same time, Bai et al. demonstrated that PFS and OS were significantly improved in EBV-positive patients treated with ICI. However, contradictory results from small cohorts and the low prevalence of EBV-positive gastric cancer have limited clear conclusions (86–88). Furthermore, differences among detection techniques, such as PCR, *in situ* hybridization, and sequencing, complicate clinical translation (89).

### 5.6 Toward a practical biomarker-guided clinical framework

Given the complexity and heterogeneity of immunotherapy-related biomarkers in advanced gastric cancer, a stepwise and clinically pragmatic approach is required. Currently, mismatch repair/microsatellite instability status should be prioritized as the initial biomarker due to its substantial predictive value and availability across most pathology laboratories. PD-L1 combined positive score may further refine patient selection, particularly in microsatellite-stable disease. Tumor mutational burden and Epstein–Barr virus status provide additional stratification, but they are best interpreted in conjunction with established biomarkers.

Emerging biomarkers related to the tumor microenvironment, such as immune cell infiltration patterns, tertiary lymphoid structures, and interferon-γ–associated gene signatures, hold promise for further optimizing immunotherapy strategies but remain investigational. Circulating tumor DNA may complement tissue-based testing by enabling real-time monitoring of treatment response and resistance. Significantly, biomarker selection should be adapted to local resource availability, balancing predictive accuracy with feasibility to ensure equitable clinical application.

**Figure 3:**
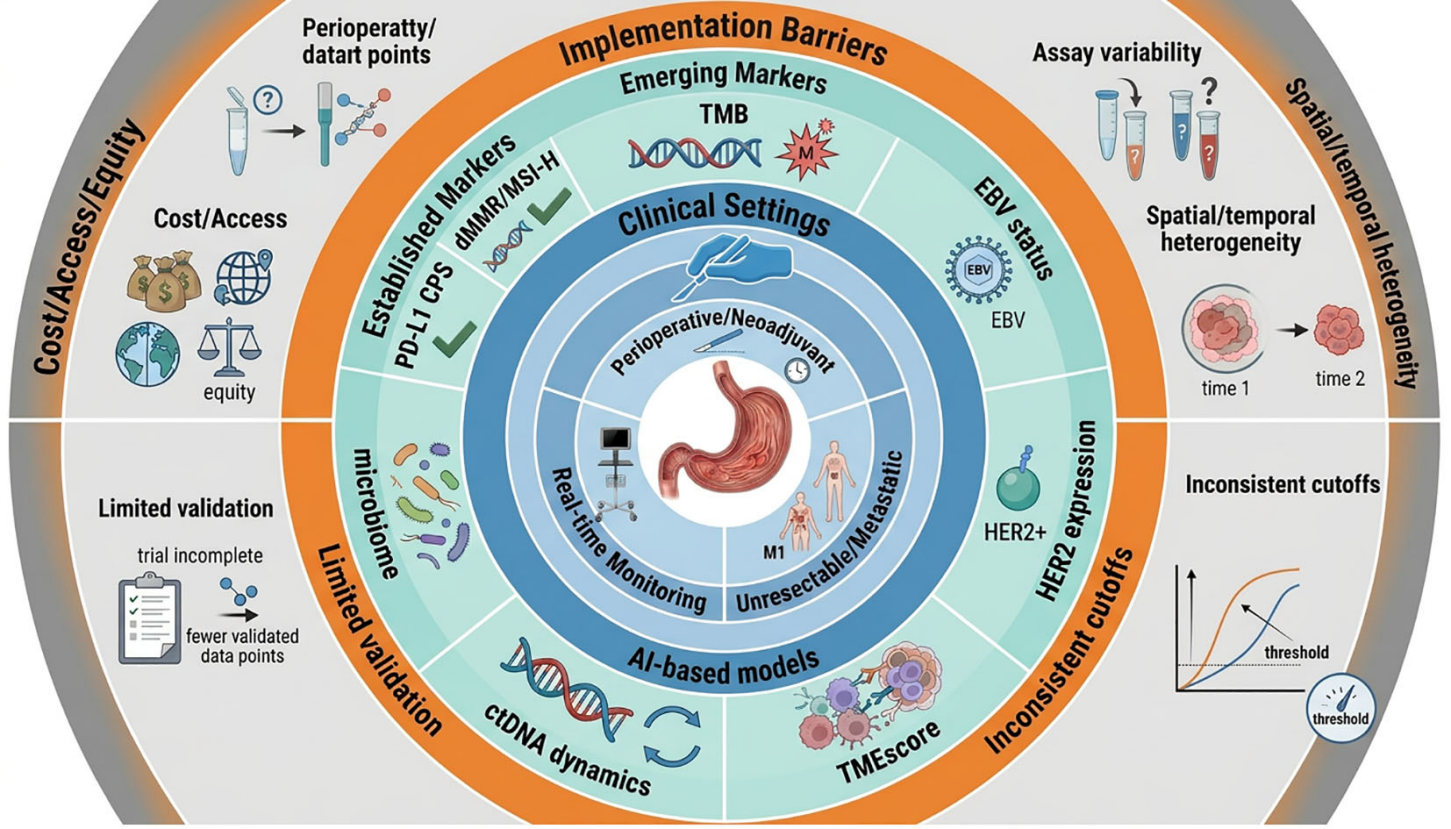
Integrated biomarker framework and implementation barriers for immunotherapy in gastric cancer.

A practical biomarker-guided framework should therefore be interpreted as a tiered decision pathway rather than a single-test algorithm. In routine clinical practice, MSI/MMR testing and PD-L1 CPS remain the most immediately actionable tools because they are relatively accessible and directly linked to ICI decision-making. HER2 testing is essential when chemoimmunotherapy is combined with trastuzumab-based targeted therapy. At the same time, TMB, EBV status, TMEscore, ctDNA dynamics, microbiome features, and AI-based models should currently be regarded as complementary or investigational markers unless supported by validated assays and treatment-specific evidence. This distinction is important because some biomarkers overlap biologically, particularly TMB-H with MSI-H, while assay variability, dynamic expression, unclear cutoffs, or insufficient prospective validation limit others. Thus, optimization of immunotherapy in gastric cancer requires integrated biomarker interpretation that considers clinical setting, treatment line, assay availability, molecular subtype, immune contexture, and real-world feasibility. A tiered framework integrating established biomarkers, emerging markers, clinical settings, and implementation barriers is presented in **Figure 3**.

The figure summarizes established and emerging biomarkers across perioperative, metastatic, and real-time monitoring settings. It also highlights major clinical translation barriers, including assay variability, spatial and temporal heterogeneity, inconsistent cutoffs, limited validation, cost, access, and equity issues.

## 6 Emerging and exploratory biomarkers

Although there is an increasing number of biomarkers guiding immunotherapy for advanced gastric cancer (AGC), a large proportion of patients still exhibit heterogeneous responses. This has led to growing interest in emerging and exploratory biomarkers that reflect the dynamic interaction between tumors and the host immune system, as well as the real-time evolution of tumors during treatment.

### 6.1 Immune landscape of the tumor microenvironment

The tumor microenvironment (TME) plays a pivotal role in determining sensitivity or resistance to immune checkpoint inhibitors (ICIs) (90, 91). In addition to the intrinsic characteristics of tumors, the cellular composition, spatial organization, and functional status of immune cells in TME also have essential effects on anti-tumor immunity. “Hot” TME to enrich the CD8+ + cytotoxic T lymphocytes, activation of dendritic cells, and Th1 cytokines polarization characteristics of signal, and usually associated with good immune treatment outcomes, and “cold” or immunosuppression TME shows limited immunity infiltration and poor reactivity. Systemic inflammatory markers that reflect the immune environment have also received attention. The neutrophil-to-lymphocyte ratio (NLR) increases, and the use of ICIs in patients with poor prognosis and immune-related adverse events also rises, potentially promoting tumor growth and reflecting the state of inflammation and adaptive immunity (92). However, evidence specific to AGC remains limited, and optimal cutoff values for clinical application have not been standardized.

In addition to cellular markers, immune-related gene expression signatures have emerged as promising predictors of the benefit from immunotherapy. In particular, gene expression profiles associated with interferon-γ (IFN-γ) signaling, antigen presentation, and T-cell activation have demonstrated predictive value in gastric cancer patients receiving ICIs (93). These features may capture functional immune activity more thoroughly than individual biomarkers such as PD-L1 expression. In addition, multiple immunohistochemical and transcriptomic studies improve the characterization of immune cell interactions and the TME internal position, which may improve patient stratification in the future.

### 6.2 Circulating tumor DNA

Circulating tumor DNA (ctDNA) is a minimally invasive, highly sensitive method for dynamic monitoring of tumors. In gastric cancer, baseline ctDNA levels and early changes during treatment have been proven to be associated with objective response, progression-free survival, and overall survival in patients receiving immunotherapy (94). Rapid clearance of ctDNA, or a significant early reduction, may indicate effective immune-mediated tumor control, whereas persistent or elevated ctDNA levels typically precede radiological progression.

Compared with imaging-based evaluations, ctDNA has several advantages. A tiny residual tumor load can be assessed in real time, enabling earlier detection of the disease and providing insight into molecular evolution during treatment and resistance mechanisms from a molecular perspective. Importantly, ctDNA analysis may help distinguish genuine from false progress, a phenomenon occasionally observed during immunotherapy that complicates radiological interpretation. In addition, ctDNA analysis can simultaneously assess genomic alterations, including tumor mutation burden, microsatellite instability, and emerging resistance mutations, thereby enhancing its efficacy as a composite biomarker. Despite the technical challenges, costs, and standardization of testing that currently limit the clinical application of ctDNA testing, AGC ctDNA immune auxiliary treatment decisions and complementary biomarkers have significant potential.

### 6.3 ctDNA dynamics and AI-based biomarker integration: clinical readiness and limitations

**Figure 4:**
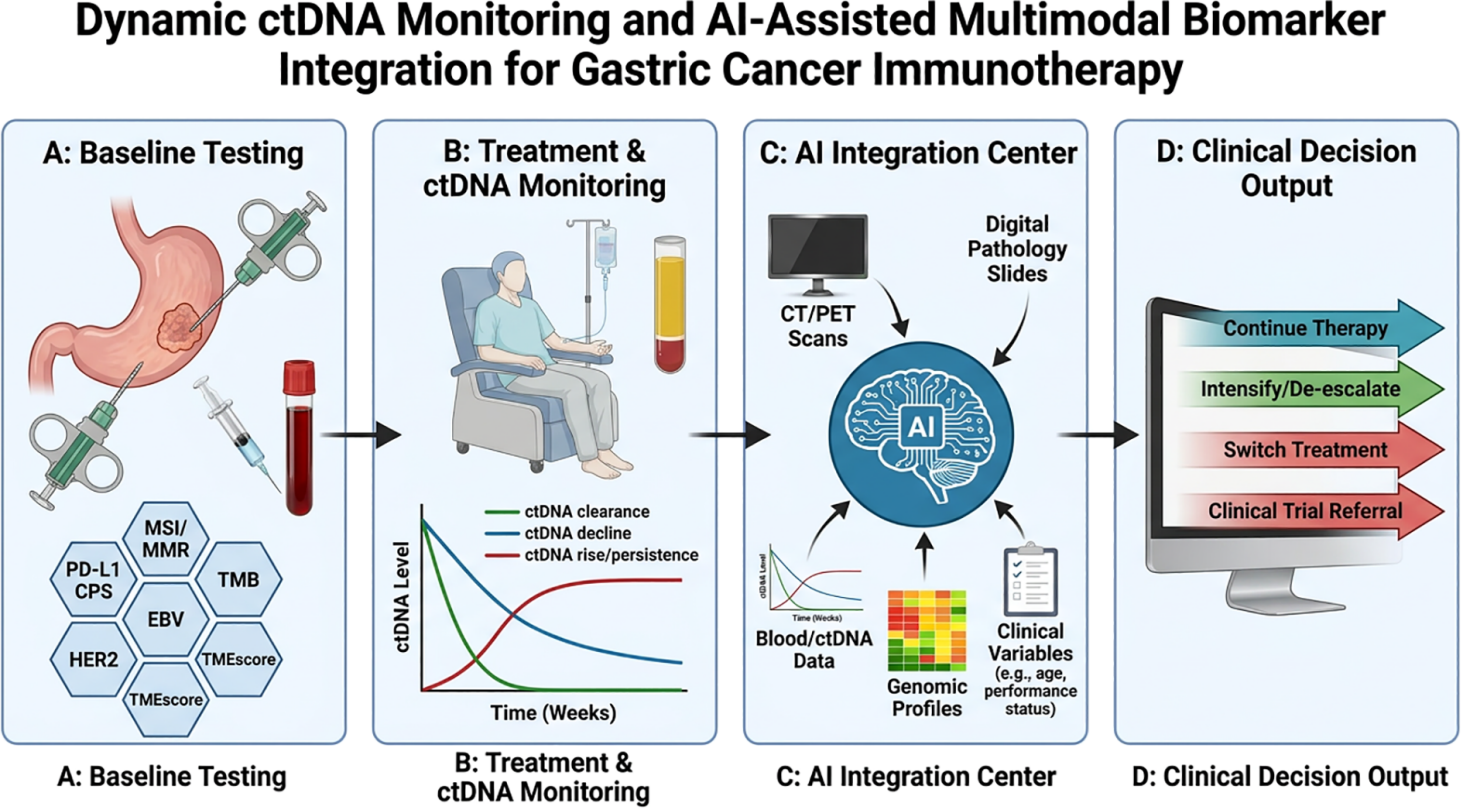
Dynamic ctDNA monitoring and AI-assisted biomarker integration in gastric cancer.

Recent gastric cancer-specific evidence suggests that ctDNA dynamics may serve as an early, clinically informative marker of ICI benefit, particularly when measured longitudinally rather than at a single baseline time point. In patients with advanced gastric cancer receiving first-line PD-1 inhibitors plus chemotherapy, early ctDNA response after two treatment cycles was associated with a higher objective response rate and longer survival, suggesting that molecular response may precede or complement radiological assessment (56). Similarly, in advanced gastric or gastroesophageal junction adenocarcinoma treated with chemotherapy plus immune checkpoint inhibition, the PRODIGE 59-FFCD 1707-DURIGAST translational analysis showed that high baseline ctDNA concentration was associated with poorer survival and that an early ctDNA decrease during treatment strongly predicted improved response, progression-free survival, and overall survival (57). In HER2-negative advanced gastric cancer, ctDNA mutation signatures and on-treatment ctDNA kinetics have also been linked to clinical benefit and resistance during immune checkpoint blockade, further supporting ctDNA as a dynamic biomarker for treatment monitoring rather than only a static genomic profiling tool (95). However, ctDNA-guided immunotherapy remains translational rather than fully established because assays differ in tumor-informed versus tumor-naïve design, sequencing depth, methylation-based versus mutation-based detection, sampling time points, response thresholds, and sensitivity in low-shedding disease. Therefore, ctDNA dynamics should currently be interpreted as a complementary biomarker that may help identify early non-responders, monitor molecular resistance, and support treatment adaptation. However, prospective trials are still needed before ctDNA can routinely guide escalation, de-escalation, or switching of ICI-based therapy. Artificial intelligence-based biomarker models may further improve prediction by integrating radiology, digital pathology, clinical variables, and molecular data. For example, deep learning models using H&E-stained whole-slide images have shown potential for predicting response to first-line PD-1 blockade in advanced gastric cancer, and multimodal radiopathomics models combining CT and pathology features have reported promising performance for predicting response to immunotherapy-based combination therapy (96, 97). Nevertheless, most AI-based biomarkers remain in the research or retrospective validation stage and are not yet ready to replace validated clinical biomarkers. Major barriers include limited external validation, small or single-region training datasets, scanner and staining variability, differences in imaging protocols, risk of overfitting, limited interpretability, lack of predefined clinical cutoffs, data privacy concerns, and the absence of prospective interventional trials showing that AI-guided treatment decisions improve patient outcomes. Accordingly, the most clinically realistic near-term role of AI is not as a stand-alone decision-maker but as an integrative risk-stratification tool that combines ctDNA dynamics, MSI/MMR status, PD-L1 CPS, TMB, EBV status, HER2 status, and TME features to improve biomarker-guided immunotherapy selection in gastric cancer. The proposed integration of longitudinal ctDNA monitoring with AI-assisted multimodal prediction is shown in **Figure 4**.

The figure illustrates how longitudinal ctDNA monitoring and AI-based multimodal analysis may support the optimization of immunotherapy in gastric cancer. By integrating ctDNA kinetics with imaging, pathology, molecular biomarkers, and clinical data, this approach may improve response assessment, resistance detection, and treatment adaptation.

## 7 Clinical applications and current limitations of immunotherapy biomarkers

### 7.1 Clinical applications

The identification of immunotherapy-related biomarkers has significantly advanced the clinical management of advanced gastric cancer (AGC), enabling more precise patient stratification and treatment decisions. Biomarkers such as PD-L1 expression, mismatch repair defects (dMMR), microsatellite instability (MSI-H), and high tumor mutational burden (TMB) are now commonly incorporated into clinical workflows to guide the use of immune checkpoint inhibitors (ICIs). In particular, MSI-H/dMMR status has become a reliable predictor of durable response to ICIs and is recognized in major clinical guidelines as a key treatment option. In addition to single biomarkers, increasing attention is being devoted to complex, environment-dependent indicators, including tumor-infiltrating lymphocytes, tertiary lymphoid structures, immune gene expression signatures (such as interferon-γ-related pathways), and tumor microenvironment (TME)-based scoring systems. These markers offer a deeper understanding of the tumor’s immune status, which helps improve prognosis assessment. Circulating biomarkers, such as circulating tumor DNA (ctDNA), can further inform the implementation of immunotherapy by providing information on treatment response and disease burden, and enable noninvasive, real-time monitoring of molecular evolution. Emerging integrated approaches that combine genomics, transcriptomics, immunophenotypes, and clinical data (typically supported by artificial intelligence (AI) and machine learning) are increasingly being explored to develop predictive models that outperform individual biomarkers. Overall, these advancements are reshaping immunotherapy, shifting it from a trial-and-error strategy to a more personalized, biomarker-driven treatment model for gastric cancer.

### 7.2 Current limitations and access barriers

Despite substantial progress, limitations have prevented the widespread and consistent clinical application of immunotherapy-related biomarkers in gastric cancer. First, significant intra- and inter-tumor heterogeneity leads to variability in biomarker expression, thereby reducing predictive accuracy when relying on a single marker. Spatial and temporal heterogeneity further complicates interpretation, as the state of the biomarkers in the primary tumor and metastatic site, as well as treatment, differs across time points. Secondly, the inconsistency in methods remains a significant challenge. Detection platforms, global thresholds, the scoring system (such as PD-L1 CPS), and differences across sequencing groups lead to inconsistent biomarker classification. This lack of standardization is particularly evident in TMB, MSI, and emerging transcriptomic features, which limits their reproducibility and comparability across disciplines. Thirdly, although composite and AI-based models show promise, most remain in the exploratory or retrospective validation stage. Limited prospective clinical validation, high costs, and technical complexity have restricted their routine clinical application. Furthermore, in the healthcare system, access to advanced molecular testing and liquid biopsy platforms remains uneven, which may exacerbate the gap in precision oncology. Finally, immune-related adverse events and unpredictable resistance mechanisms underscore the need for biomarkers that can predict not only the reaction but also toxicity and long-term outcomes. Addressing these limitations is critical to translating biomarker discoveries into robust clinical tools.

### 7.3 Global access, cost constraints, and equity considerations

Despite the clinical promise of biomarker-guided immunotherapy, its real-world implementation remains uneven across healthcare systems, particularly in low- and middle-income countries (LMICs). Limited access to advanced molecular diagnostics, high costs of immune checkpoint inhibitors, and insufficient reimbursement policies pose significant barriers to equitable cancer care. Comprehensive biomarker testing, such as next-generation sequencing–based tumor mutational burden assessment or multiplex immunohistochemistry, is often unavailable or unaffordable in resource-constrained settings, restricting patient eligibility for immunotherapy.

Simplified and cost-effective diagnostic strategies may help bridge this gap. For example, immunohistochemistry-based mismatch repair (MMR) testing and PD-L1 scoring represent relatively accessible tools that can guide treatment decisions without requiring complex infrastructure. In addition, circulating tumor DNA–based assays offer a minimally invasive alternative for disease monitoring and response assessment, with potential advantages in settings where repeated tissue biopsies are impractical. However, broader clinical validation and cost reduction are necessary before widespread adoption can occur.

Addressing global disparities in immunotherapy access will require coordinated efforts encompassing assay standardization, resource-adapted clinical algorithms, and health policy initiatives to improve affordability and reimbursement. Integrating real-world data from diverse geographic regions will be essential to developing sustainable, globally applicable immunotherapy strategies for gastric cancer.

## 8 Conclusions and future prospects

Immunotherapy has transformed the management of advanced gastric and gastroesophageal junction cancer, but its clinical value depends on accurate patient selection, rational combination strategies, and realistic translation into routine care. The updated evidence reviewed here indicates that biomarker-guided immunotherapy should be understood as an integrated decision framework rather than a single-marker approach. MSI/MMR status is currently among the most clinically actionable biomarkers, particularly because dMMR/MSI-H tumors exhibit strong biological and clinical sensitivity to immune checkpoint blockade. PD-L1 CPS remains important for enriching patient selection in metastatic chemoimmunotherapy, but its interpretation is limited by assay variability, tumor heterogeneity, dynamic expression, and inconsistent thresholds across trials. TMB, although biologically attractive, requires gastric cancer-specific caution because its predictive value overlaps substantially with MSI-H biology and lacks a universally validated disease-specific cutoff. EBV positivity, HER2 status, immune-cell infiltration, TMEscore, ctDNA dynamics, and AI-assisted multimodal models further expand the biomarker landscape. Still, these markers should be interpreted in the context of the clinical setting, assay readiness, validation strength, and treatment context.

Future progress will depend on aligning biomarker development with the evolving clinical use of immunotherapy across metastatic, perioperative, and neoadjuvant settings. In resectable disease, early molecular testing at diagnosis is increasingly important because perioperative and neoadjuvant ICI trials suggest that selected patients, particularly those with dMMR/MSI-H or immune-inflamed tumors, may benefit from immune-based intensification or treatment de-escalation strategies. In metastatic disease, biomarker interpretation must account for combination regimens, including chemotherapy plus PD-1/PD-L1 blockade and HER2-targeted chemoimmunotherapy. Beyond static tissue biomarkers, longitudinal ctDNA dynamics may offer earlier insight into molecular response, residual disease, clonal evolution, and resistance. At the same time, TMEscore and spatial immune profiling may better capture the immune contexture that determines ICI sensitivity. AI-based models integrating digital pathology, radiology, molecular data, and clinical variables may eventually support risk stratification. Still, their current role remains investigational, as most models lack broad external validation, prospective testing, predefined clinical cutoffs, and demonstrated impact on treatment decisions.

To move biomarker-guided immunotherapy from scientific promise to clinical reality, future research should prioritize prospective validation, harmonized assay platforms, gastric cancer-specific cutoffs, standardized reporting, and clinically interpretable composite models. Equally important is the development of resource-adapted biomarker algorithms that can be implemented across diverse healthcare systems. In routine practice, accessible tools such as MMR immunohistochemistry, MSI testing, HER2 assessment, and PD-L1 CPS should remain central to treatment decision-making. At the same time, TMB, EBV status, TMEscore, ctDNA, microbiome markers, and AI-based models should be incorporated cautiously as complementary tools, provided validated assays support them and are locally feasible. Ultimately, optimizing immunotherapy in gastric cancer will require the coordinated integration of tumor biology, the immune microenvironment, dynamic disease monitoring, clinical trial evidence, and equitable access. Such a balanced, evidence-based approach can improve patient selection, reduce unnecessary treatment exposure, support rational combination therapy, and advance precision immunotherapy from selective benefit to a more reliable, globally accessible standard of care.
